# Unleashing the potential of mRNA therapeutics for inherited neurological diseases

**DOI:** 10.1093/brain/awae135

**Published:** 2024-04-25

**Authors:** Edoardo Monfrini, Giacomo Baso, Dario Ronchi, Megi Meneri, Delia Gagliardi, Lorenzo Quetti, Federico Verde, Nicola Ticozzi, Antonia Ratti, Alessio Di Fonzo, Giacomo P Comi, Linda Ottoboni, Stefania Corti

**Affiliations:** Neurology Unit, Fondazione IRCCS Ca’ Granda Ospedale Maggiore Policlinico, Milan 20122, Italy; Department of Pathophysiology and Transplantation (DEPT), Dino Ferrari Centre, University of Milan, Milan 20122, Italy; Department of Pathophysiology and Transplantation (DEPT), Dino Ferrari Centre, University of Milan, Milan 20122, Italy; Neurology Unit, Fondazione IRCCS Ca’ Granda Ospedale Maggiore Policlinico, Milan 20122, Italy; Department of Pathophysiology and Transplantation (DEPT), Dino Ferrari Centre, University of Milan, Milan 20122, Italy; Department of Pathophysiology and Transplantation (DEPT), Dino Ferrari Centre, University of Milan, Milan 20122, Italy; Stroke Unit, Fondazione IRCCS Ca’ Granda Ospedale Maggiore Policlinico, Milan 20122, Italy; Department of Pathophysiology and Transplantation (DEPT), Dino Ferrari Centre, University of Milan, Milan 20122, Italy; Neurology Unit, Fondazione IRCCS Ca’ Granda Ospedale Maggiore Policlinico, Milan 20122, Italy; Department of Pathophysiology and Transplantation (DEPT), Dino Ferrari Centre, University of Milan, Milan 20122, Italy; Department of Neurology, Laboratory of Neuroscience, IRCCS Istituto Auxologico Italiano, Milan 20149, Italy; Department of Pathophysiology and Transplantation (DEPT), Dino Ferrari Centre, University of Milan, Milan 20122, Italy; Department of Neurology, Laboratory of Neuroscience, IRCCS Istituto Auxologico Italiano, Milan 20149, Italy; Department of Neurology, Laboratory of Neuroscience, IRCCS Istituto Auxologico Italiano, Milan 20149, Italy; Department Medical Biotechnology and Translational Medicine, University of Milan, Milan 20100, Italy; Neurology Unit, Fondazione IRCCS Ca’ Granda Ospedale Maggiore Policlinico, Milan 20122, Italy; Neurology Unit, Fondazione IRCCS Ca’ Granda Ospedale Maggiore Policlinico, Milan 20122, Italy; Department of Pathophysiology and Transplantation (DEPT), Dino Ferrari Centre, University of Milan, Milan 20122, Italy; Department of Pathophysiology and Transplantation (DEPT), Dino Ferrari Centre, University of Milan, Milan 20122, Italy; Department of Pathophysiology and Transplantation (DEPT), Dino Ferrari Centre, University of Milan, Milan 20122, Italy; Department of Neuroscience, Neuromuscular and Rare Diseases Unit, Fondazione IRCCS Ca’ Granda Ospedale Maggiore Policlinico, Milan 20122, Italy

**Keywords:** personalized medicine, monogenic disorders, mRNA, neurological diseases

## Abstract

Neurological monogenic loss-of-function diseases are hereditary disorders resulting from gene mutations that decrease or abolish the normal function of the encoded protein. These conditions pose significant therapeutic challenges, which may be resolved through the development of innovative therapeutic strategies. RNA-based technologies, such as mRNA replacement therapy, have emerged as promising and increasingly viable treatments. Notably, mRNA therapy exhibits significant potential as a mutation-agnostic approach that can address virtually any monogenic loss-of-function disease.

Therapeutic mRNA carries the information for a healthy copy of the defective protein, bypassing the problem of targeting specific genetic variants. Moreover, unlike conventional gene therapy, mRNA-based drugs are delivered through a simplified process that requires only transfer to the cytoplasm, thereby reducing the mutagenic risks related to DNA integration. Additionally, mRNA therapy exerts a transient effect on target cells, minimizing the risk of long-term unintended consequences. The remarkable success of mRNA technology for developing coronavirus disease 2019 vaccines has rekindled interest in mRNA as a cost-effective method for delivering therapeutic proteins. However, further optimization is required to enhance mRNA delivery, particularly to the CNS, while minimizing adverse drug reactions and toxicity.

In this comprehensive review, we delve into past, present and ongoing applications of mRNA therapy for neurological monogenic loss-of-function diseases. We also discuss the promises and potential challenges presented by mRNA therapeutics in this rapidly advancing field. Ultimately, we underscore the full potential of mRNA therapy as a game-changing therapeutic approach for neurological disorders.

## Teaser

The success of COVID-19 mRNA vaccines has rekindled interest in mRNA as a cost-effective means of delivering therapeutic proteins. Monfrini *et al.* explore potential applications of mRNA therapies for neurological monogenic loss-of-function diseases, and highlight some of the possibilities and challenges in this rapidly advancing field.

## Introduction

Neurological monogenic loss-of-function diseases are genetic disorders caused by mutations in a single gene that abolish or reduce the function of the encoded protein. Notable examples include autosomal recessive disorders (e.g. spinal muscular atrophy,^1^ neuronopathic Gaucher disease,^2^ Tay-Sachs disease^3^ and phenylketonuria^4^); X-linked diseases (e.g. Duchenne muscular dystrophy^5^ and Fragile X syndrome^6^); and autosomal dominant disorders due to haploinsufficiency (e.g. optic atrophy 1 due to *OPA1* mutations^7^ and GRN-associated frontotemporal dementia^8^). Although these individual diseases have low prevalence rates, they collectively affect millions of individuals worldwide.^9-11^

The deleterious effects of genetic mutations in loss-of-function diseases can theoretically be corrected by delivering mRNA to restore healthy protein production and prevent dysfunctional molecular cascades induced by lost protein function.^12^ Although most research has focused on gene therapy methods, including gene transfer and editing, RNA-based therapies have emerged as equally promising approaches that may offer advantages over more invasive or permanent DNA-based procedures.^13,14^

The seminal 1978 work by Grunhaus and Horwitz marked a pivotal moment in mRNA therapy development, demonstrating that exogenous isolated synthetic mRNA can induce protein expression *in vitro*.^12,15^ In 1990, Wolf *et al*.^16^ made a significant breakthrough by injecting mRNA into mouse skeletal muscle, providing the first convincing evidence of successful mRNA delivery and subsequent transitory production of specific proteins *in vivo*. In the early 1990s, researchers explored mRNA use as a potential treatment or vaccination strategy against cancer and infectious disorders, but its *in vivo* application was constrained by its inflammatory nature.^17-20^ The field changed in 2005, when Karikó and Weissman^19^ revealed that nucleoside modification (substitution of pseudouridine for uridine) profoundly impacted mRNA immunogenicity and stability.^17,21^ In 2023, they were collectively awarded the Nobel Prize in Physiology or Medicine for their pioneering research on base modifications, which was pivotal in enabling the development of mRNA vaccines against coronavirus disease 2019 (COVID-19; https://www.nobelprize.org/prizes/medicine/2023/).

## Advantages and drawbacks of mRNA-based therapeutics

The unique ability of mRNA to allow transient protein expression makes it an attractive tool for a wide range of research and clinical applications, including vaccine development, therapeutic mRNA protein replacement, genome editing, cell reprogramming and cell therapy.^22,23^

Compared to conventional treatments, mRNA therapy offers several benefits. As mRNA is translated within the cell, the encoded proteins can address the intracellular compartment, including cellular membranes. This enables direct targeting of several metabolic diseases that are difficult to treat with recombinant proteins, which can exert their effects mainly in the extracellular fluid.^24^ Additionally, mRNA-based therapies may offer advantages over DNA-based therapies,^25^ primarily because mRNA can avoid some biological processes, e.g. transcription and insertion into the nucleus. Therapeutic mRNA can be immediately translated in cytoplasm without moving through the nucleus, and in non-dividing cells, such as neurons. Non-integrating episomal self-organization via adeno-associated virus (AAV) is currently the preferred technology for DNA-based therapies; however, some integrating vectors are used in clinical practice.^26,27^ Therefore, mRNA-based treatments offer increased safety because they do not integrate into the host genome, reducing the risk of mutagenic activity. Moreover, mRNA-based treatments have a shorter duration of action, providing additional safety and control.^28-30^ Another advantage is the fast translation of mRNA into the corresponding protein. Although most monogenic loss-of-function disorders are chronic, the rapid expression (hours compared to days with DNA-based delivery) of a gene of interest could quickly achieve activation or inhibition of a target pathway, providing immediate therapeutic effect while more durable or permanent therapies are administered.^31,32^

Although mRNA therapy shows great promise for protein replacement, it has some disadvantages. The most significant challenge is the potential for unwanted immune-mediated toxicity. Unmodified RNA molecules can enter cells and activate signalling receptors, including endosomal Toll-like receptors (TLR) 3, 7 or 8.^33,34^ The risk of immune response can be reduced by modifying the mRNA structure via incorporation of modified nucleosides, such as pseudouridine; altering the 5′ cap structure, including poly(A) tails; and optimizing the delivery method.^17-19,35^ These modifications can increase mRNA stability and decrease the chance of RNase cleavage, thereby decreasing cytokine-mediated toxicity from non-specific immune reactivity. Notably, the administration of mRNA COVID-19 vaccines to hundreds of millions of subjects worldwide demonstrates the substantial safety of mRNA encapsulated in lipid nanoparticles (LNPs). Although advancements in RNA chemistry have dramatically reduced the immunogenicity of RNA products, the long-term effects of RNA administration on immunity responses remain unexplored. The second major drawback of mRNA therapy is the need for repeated administration to ensure prolonged therapeutic protein expression in target tissues. For monogenic disorders, the doses needed to preserve therapeutic efficacy directly depends on RNA stability and protein turnover. In most preclinical studies of mRNA therapy for monogenic disorders, protein expression was monitored over months, with an interval of weeks between repeat injections. Therapies necessitating multiple doses tend to be less feasible and more expensive compared to one-shot approaches. Repeated doses pose practical concerns especially in neurological patients, who may exhibit motor and cognitive disabilities.

## Pharmacology of therapeutic mRNA

**Figure 1:**
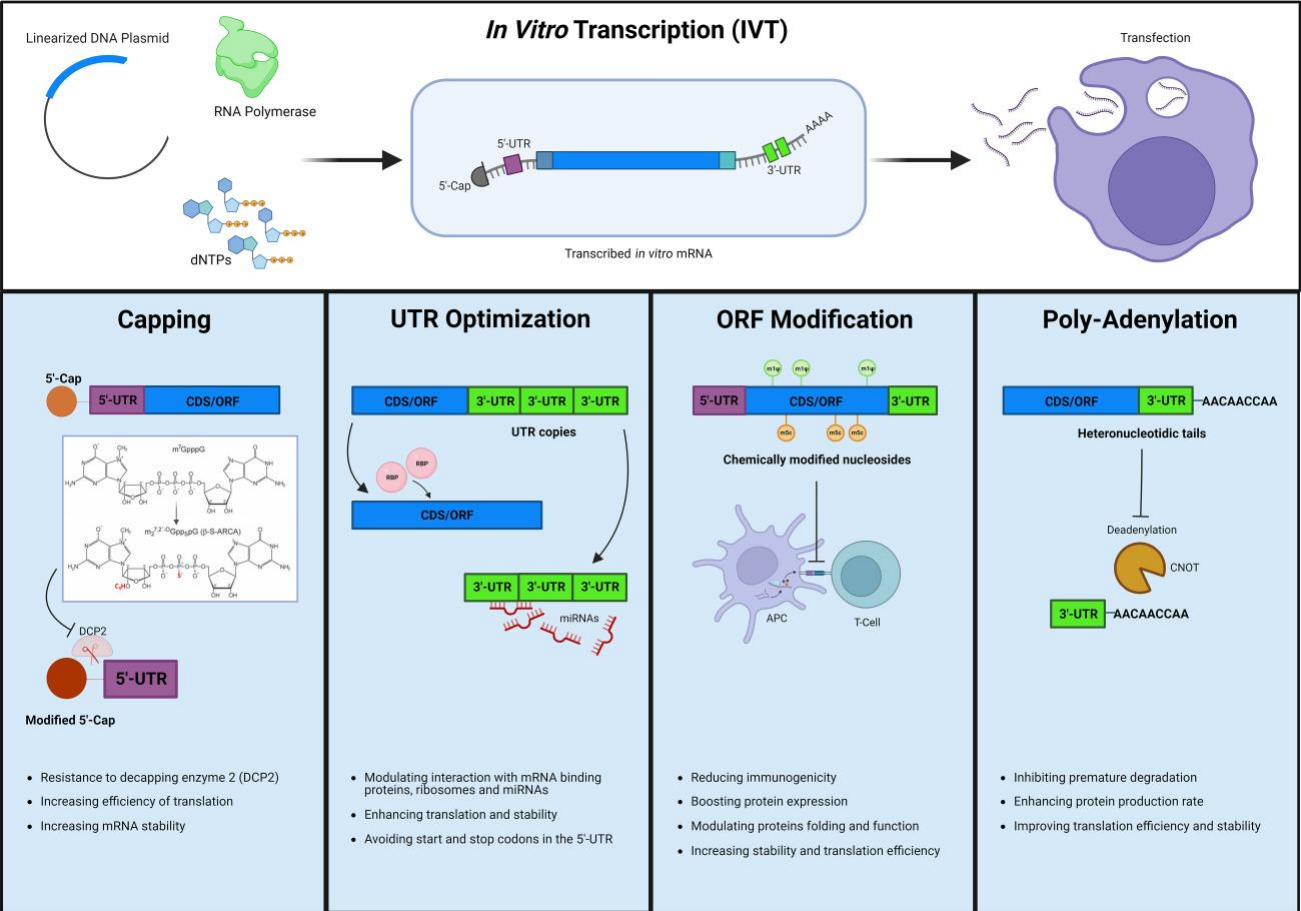
**Design and structure of therapeutic mRNA.** Graphic representation of the basic elements of the therapeutic mRNA synthesis, design and modifications, including capping, untranslated region (UTR) optimization, open reading frame (ORF) modifications and poly-adenylation. Created with BioRender.com.

**Figure 2:**
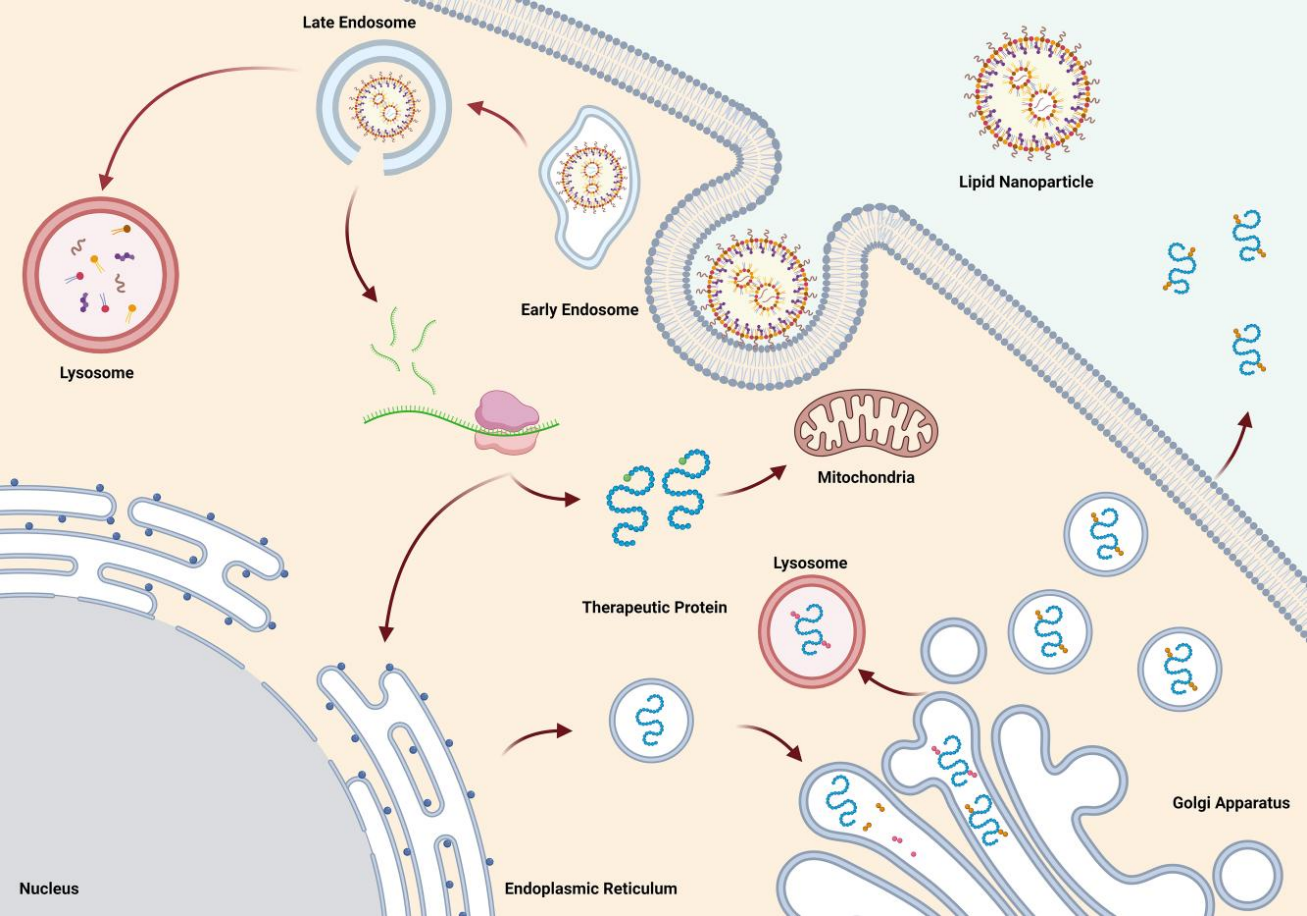
**Cellular metabolism of therapeutic mRNA.** Lipid nanoparticles deliver therapeutic mRNA into the cells. The mRNA released from endosomes (‘endosomal escape’) is translated by ribosomes into therapeutic protein. Targeting sequences or post-translational modifications help the protein reach its intracellular targets (e.g. cytoplasm, mitochondrion, lysosome, etc.) or extracellular targets. Created with BioRender.com.

Therapeutic mRNA mimics the structure of naturally occurring mature and processed mRNA in the cytoplasm of eukaryotic cells. Mature mRNA is single-stranded, with a 5′ cap and 3′ poly(A) tail. Start and stop codons define the open reading frame (ORF) of the relevant gene, which is surrounded by untranslated regions (UTRs) (Fig. 1). Any mRNA molecule can be synthesized through *in vitro* transcription using phage RNA polymerases in a cell-free system,^36^ enabling mRNA generation from an encoding plasmid, unlocking immense possibilities for customized mRNA development (Fig. 2).^37^

Therapeutic mRNA primarily exhibits pharmacodynamic action in cytoplasm,^33,38^ requiring successful cellular uptake, intracellular release (endosomal escape) and translation into protein (Fig. 2). Cytoplasmic bioavailability of mRNA is limited by two factors: rapid degradation by extracellular RNases; and the inability of large negatively charged mRNA molecules to passively diffuse through cell membranes.^38^ Eukaryotic cells can actively uptake naked mRNA but often with low uptake rate and cytosolic transfer.^39^

Nanoparticles can deliver therapeutic mRNA, enabling its internalization into cells through the endosomal system. Cationic nanoparticles are commonly employed because they encapsulate negatively charged mRNA. An ideal nanoparticle possesses appropriate charge density and molecular weight; low toxicity and immunogenicity; and desirable pharmacokinetic parameters (e.g. dispersion, biodegradation and clearance capacity).^40^ LNPs, characterized by an aqueous core and lipid bilayer shell, have emerged as efficient tools for mRNA encapsulation and delivery.^41^ LNPs typically comprise four specific lipids: an ionizable or cationic lipid for RNA loading; helper or neutral lipids as the particle base; cholesterol for particle stabilization; and lipids attached to polyethylene glycol (PEG) to improve stability, prevent lipid aggregation and shield the particle charge.^42^

The pharmacokinetics of mRNA-based treatments are largely determined by the mRNA and encoded protein half-lives. Complex cellular processes influence mRNA pharmacology. The bioactive protein is modified by post-translational changes, and signal peptides targeting a particular cell compartment (e.g. mitochondria) determine its final destination. Secretion of the translated protein into extracellular medium or circulation enables therapeutic effects in neighbouring cells or distant organs.^43^

## mRNA therapy for neurological diseases

**Figure 3:**
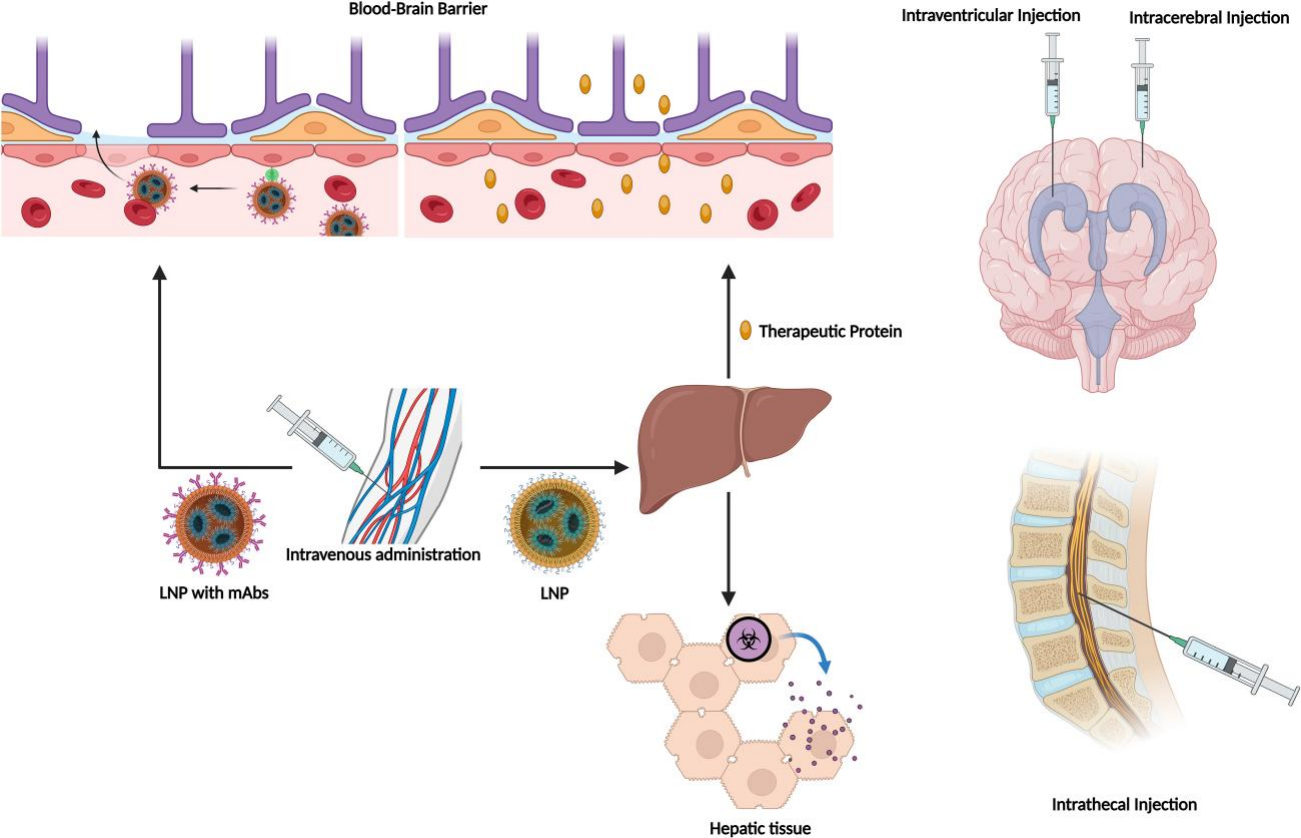
**mRNA-based therapeutic approaches for neurological disorders.** For treatment of neurological disorders, therapeutic mRNA can be administered by intravenous or intrathecal routes. Intravenous administration is preferable for neurological diseases associated with systemic metabolic defects that can be addressed by targeting the liver, and for reducing the build-up of neurotoxic substances. On the other hand, intrathecal/intracerebral/intraventricular administrations may be preferable in neurological disorders requiring direct pharmacological engagement of the CNS. Modified lipid nanoparticles (LNPs) that can be intravenously administered, and cross the blood–brain barrier, are under development. mAbs = monoclonal antibodies. Created with BioRender.com.

Clinical applications of mRNA replacement therapy for neurological diseases comprise two broad categories: neurological diseases associated with systemic metabolic defects that can be addressed by targeting the liver; and neurological disorders caused by nervous system defects that require direct pharmacological engagement of nervous tissues (Fig. 3). Preclinical data are accumulating in both settings, whereas clinical studies have predominantly focused on the first group. Relevant examples are described later (Supplementary Table 1).

### Methylmalonic acidaemia/aciduria

Methylmalonic acidaemia (MMA) is a rare severe autosomal recessive disorder caused by deficient activity of methylmalonyl-CoA mutase (MUT), the mitochondrial enzyme essential for the breakdown of branched-chain amino acids. The resulting accumulation of harmful levels of methylmalonic acid and other metabolites causes metabolic acidosis, neurological impairment and developmental delay,^44^ with potential long-term severe neurological complications and chronic renal failure. Enzyme replacement therapy (ERT) remains unavailable.^44,45^ Current therapeutic approaches involve strict dietary restrictions, supplements (e.g. carnitine) and antibiotics and supportive measures. In severe cases, elective liver transplantation can boost enzyme activity, and liver-kidney transplantation may be considered for renal insufficiency.^44^

Systemic mRNA therapy is a potential treatment for MUT enzyme deficiency. Venditti’s group and Moderna investigated the administration of mRNA encoding wild-type MUT to fibroblasts from MMA patients with complete enzymatic deficiency (mut0), wild-type mice and MMA mouse models with MUT deficiency. Researchers optimized an mRNA with 5-methoxyU and encapsulated it in LNPs.^44-47^  *In vitro* analysis in fibroblasts of mut0 patients indicated that the mRNA-encoded protein was enzymatically active and properly localized in mitochondria. An intravenous bolus injection of LNP-formulated human *MUT* mRNA in wild-type mice generated significant MUT enzyme as early as 2 h after injection, peaking at 16 h and remaining detectable for 7 days, resulting in decreased plasma methylmalonic acid starting 6 h after administration (maximum reduction, 75%–85%). In MMA mice, a single intravenous injection dose-dependently reduced plasma methylmalonic acid concentrations, which returned to pretreatment levels in 2 weeks. Weekly intravenous administration of *MUT* mRNA for 6 weeks improved the survival and weight of MMA mice, whereas untreated control mice died. Repeated intravenous administration caused no apparent adverse effects, including no increase of liver inflammation or toxicity markers. A clinical trial (ClinicalTrials.gov: NCT03810690) was designed to investigate the safety, pharmacokinetics and pharmacodynamics of LNP-encapsulated *MUT* mRNA (mRNA-3704) in MMA patients but was withdrawn before dosing initiation due to a business decision. An ongoing study (NCT04899310) is recruiting participants to assess the safety, pharmacokinetics and pharmacodynamics of mRNA-3705 in individuals with isolated MMA.

Overall, the preclinical evidence demonstrates that systemically administered mRNA can restore hepatic mitochondrial proteins, presenting a promising new avenue for molecular treatment of MMA and other mitochondrial diseases.

### Propionic acidaemia/aciduria

Propionic acidaemia (PA) is a severe early-onset metabolic disorder leading to neurological complications. It arises due to defects in the mitochondrial enzyme propionyl-CoA carboxylase (PCC), comprising six alpha (PCCA) and six beta (PCCB) subunits. Without treatment, hyperammonaemia, infections, cardiomyopathy and neurological issues can lead to early mortality.^48^

There are two forms of PCC deficiency: PCCA-deficient (type I) and PCCB-deficient (type II). Moderna is addressing the issue of dimeric enzyme activity restoration using dual mRNAs encoding both hPCCA and hPCCB subunits encapsulated in LNPs.^49^  *In vitro* investigations using patients’ fibroblasts demonstrated that dual mRNA encoding both PCC proteins effectively targeted mitochondria and substantially elevated PCC enzyme activity (5- to 24-fold) compared to single mRNA administration. These findings suggest that combining both PCC subunits significantly enhances PCC activity.^49^

In a hypomorphic mouse model of PA, treatment with dual LNP-encapsulated mRNAs yielded plasma ammonia reduction comparable to standard care for PA (carglumic acid) but additionally reduced other plasma primary biomarkers, indicating broader propionate metabolism restoration.^49^ Following administration of dual mRNA (single intravenous bolus), the liver exhibited significantly increased PCC protein subunits and enzyme activity within 6 h, peaking at 2 days and detectable levels were maintained for 21 days. Peak hPCCA and hPCCB concentrations were similar to endogenous protein levels in the human liver. These observations suggest that dual mRNA therapy could be a rapid and durable strategy to restore liver enzyme function during life-threatening acute metabolic decompensation. Long-term 3- and 6-month repeat-dose trials in animal models of PA (dose every 3–4 weeks) revealed dose-dependent therapeutic restoration of PCC enzyme function in the liver, including correct mitochondrial localization and sustained reduction of major disease-associated toxins, without associated adverse effects or immune-mediated reactions against the therapeutic compound. A planned phase 1 clinical trial (ClinicalTrials.gov ID: NCT04159103) is recruiting PA patients.

### Phenylketonuria

Phenylketonuria (PKU) is an autosomal recessive metabolic disorder caused by deficiency of the enzyme phenylalanine hydroxylase (PAH), which converts phenylalanine into tyrosine in the liver. If untreated, the accumulation of phenylalanine and its byproducts can cause severe neurological impairment,^50^ with clinical manifestations including progressive motor difficulties, epilepsy, microcephaly and severe intellectual disability. These symptoms can be partially prevented by early intervention with dietary restriction and medications that increase phenylalanine catabolism.^51-53^ The US Food and Drug Administration has approved two medications that lower phenylalanine levels in patients with PKU: pegvaliase, a PEGylated recombinant phenylalanine ammonia lyase (PAL) enzyme substitution therapy; and sapropterin dihydrochloride (KUVAN®), a supplemental tetrahydrobiopterin cofactor that boosts residual enzyme activity. However, each treatment is limited to a specific patient subgroup, failing to meet all patients’ needs. Moreover, strict adherence to the PKU diet is difficult, and some patients experience side effects from medication. Treated patients often experience neurological symptoms, e.g. tremors or brisk deep tendon reflexes and even progressive neurological deterioration. The long-term prognosis of treated phenylketonuria remains unsatisfactory and effective therapies are needed.^51^

A promising alternative for addressing PKU is mRNA replacement therapy.^54^ The Chivukula group^55^ introduced LNP-encapsulated full-length mRNA encoding human codon-optimized PAH (hPAH) in their proprietary lipid nanoparticle (lipid-enabled and unlocked nucleic acid modified RNA, LUNAR) designed to target hepatocytes. They administered it to wild-type mice and a PAH mouse model with severe hyperphenylalaninaemia (Pah^enu2^ model). Wild-type mice given a single intravenous dose (3 mg/kg) showed a 2-fold increase in liver PAH protein after 6 h, returning to control levels after 24 h. In the Pah^enu2^ model, a single intravenous administration of LUNAR-hPAH mRNA (10 mg/kg) restored phenylalanine catabolism, without significant adverse events. Phenylalanine levels decreased by approximately 20-fold at 24 h (similar to results with a strict diet) and returned to previous levels after 72 h. Repeated dosing (3 mg/kg every 3 days) was well-tolerated and yielded a cumulative phenylalanine-lowering effect, peaking at 6 h and ending 48 h after each dosing. Similarly, Cacicedo *et al.*^56^ experimented with the administration of mouse Pah (MmPah) mRNA via intravenous injection, using LNPs for delivery. A single injection of MmPah mRNA-LNPs significantly reduced phenylalanine in the sera, livers and brains (∼10-fold decrease at 24 h, ∼2-fold decrease at 48 h). Repeated dosing (every 5 days) was well-tolerated and reduced plasma phenylalanine to physiological levels.

The Chivukula group^54^ proposed the use of PAL mRNA as an alternative therapeutic strategy. The PAL enzyme is derived from *Anabaena variabilis* and breaks down phenylalanine into ammonia and trans-cinnamic acid. They demonstrated that LUNAR delivery technology effectively delivered bacterial PAL mRNA (avPAL) into the liver of PKU mice. They observed dose-dependent expression of enzymatically active PAL protein in the liver, which drastically reduced phenylalanine for 72 h and exerted a lower persistent effect for at least 96 h. LUNAR could also deliver a less immunogenic plant-derived PAL mRNA, which similarly impacted serum phenylalanine levels. These discoveries underscore the remarkable capability of LUNAR to target and deliver PAL mRNA to hepatic tissue. This approach replaces the faulty PAH protein, effectively reducing serum phenylalanine levels and tackling the cause of neurological symptoms in PKU.

Despite promising preclinical results, additional research must examine the safety and efficacy of mRNA therapy for PKU in humans. The previous achievements demonstrate the feasibility of mRNA-based protein replacement therapy as a prospective PKU treatment.

### Arginase deficiency

Mutations in the arginase 1 (*ARG1*) gene cause arginase deficiency, a rare autosomal recessive metabolic disorder.^57^ Arginase deficiency disrupts the urea cycle, causing ammonia accumulation and clinical manifestations (e.g. headaches, hypo/hyperventilation, seizures, coma and even death).^58^ Current treatment involves dietary modification, which mainly treats symptoms, without sufficiently preventing cognitive abnormalities.^59^

Asrani *et al*.^60^ conducted *in vitro* and *in vivo* studies of ARG1 mRNA expression and its effects on the urea cycle. They demonstrated that mRNA transfection significantly increased *in vitro* ARG1 protein expression and activity in key cell types.^60^ In HepG2 cell line and GM00954 cells (ARG1-deficient patient fibroblasts), protein expression was increased approximately 20- and 30-fold, respectively. *In vitro* testing confirmed the recombinant protein’s bioactivity. Using capillary electrophoresis, immunohistochemistry and *in situ* hybridization, they demonstrated that intravenous administration of human LNP-encapsulated ARG1 mRNA increased recombinant protein expression within the liver in wild-type mice. After a single dose of hARG1 mRNA, wild-type mice expressed ARG1 mRNA and protein for up to 168 h. Compared to the control group (eGFP mRNA), treatment increased protein expression by ∼25-fold at 24 h and ∼6-fold at 168 h.

Moderna and Truong *et al*.^61,62^ systematically administered human codon-optimized LNP-encapsulated ARG1 mRNA (in capsules, every 3 days) to a conditional knockout mouse model of ARG1 depletion. Treated animals exhibited several positive outcomes, including weight maintenance, plasma ammonia and arginine normalization, prevention of hepatic guanidinoacetic acid accumulation and hepatic arginine normalization without hepatotoxicity. Exogenous ARG1 protein was distributed throughout the liver, as under physiological conditions. Pharmacokinetic tests showed that LNPs efficiently delivered hARG1 mRNA, immediately yielding peak hARG1 levels in the liver. Translated ARG1 protein from LNP-hARG1 persisted, showing 54% of normal hepatic arginase activity after 24 h, whereas control animals showed no hepatic arginase activity and died.

The same group tested intermittent mRNA injection therapy to prevent CNS dysmyelination during early neonatal development in a severe constitutive ARG1-deficient mouse model.^63^ The wild-type Arg1^+/+^ group had the lowest G-ratio (inner to outer diameter of the myelin sheath), indicating thicker myelin layers, whereas the untreated Arg1^−/−^ group had the highest G-ratio, indicating thinner myelin layers. The G-ratio of the mRNA-treated Arg1^−/−^ group was similar to wild-type, suggesting the therapeutic potential of early daily intraperitoneal mRNA injections for mitigating CNS dysmyelination and enhancing axon myelination in arginase deficiency. Because of the relatively brief half-lives of mRNA and ARG1 protein,^64^ this therapy requires frequent injections to maintain therapeutic ARG1 activity, which is crucial for addressing oligodendrocyte dysfunction, preserving urea cycle function and ensuring survival.

Overall, these experiments show that hARG1 mRNA enclosed in liver-targeted nanoparticles can effectively treat murine arginase deficiency. The promising pre-clinical results suggest the possibility of molecular therapy for patients with arginase deficiency.

### Fabry disease

Fabry disease is a rare lysosomal storage disorder resulting from loss-of-function mutations in *GLA*, the gene encoding lysosomal enzyme alpha-galactosidase A (α-Gal A).^65^ This leads to elevated levels of two non-degraded glycosphingolipids—globotriaosylceramide (Gb3) and globotriaosylsphingosine (lyso-Gb3)—in the kidney, heart and skin. Patients experience angiokeratomas, acroparesthesias, hypohidrosis, corneal and lenticular opacities and proteinuria. Disease progression may lead to end-stage renal disease and cardiac and cerebrovascular complications.

Fabry disease is treated using ERT and chaperone therapy (CT). Gene therapy, gene editing, substrate reduction therapy (SRT) and combinations of ERT, SRT or CT are actively being explored. However, treated patients experience declining renal and cardiovascular function and ERT administration every other week is burdensome; thus, new therapies are needed.

Zhu *et al*.^66^ conducted preclinical investigations in mouse and non-human primate models of Fabry disease, using a therapeutic mRNA encoding human α-Gal A. They found that low levels of residual enzyme activity (i.e. ∼1%) were sufficient to reduce non-degraded lipid accumulation. A single intravenous infusion of LNP-encapsulated α-Gal A mRNA dose-dependently restored α-Gal A enzyme in the livers of mouse models (α-Gal A KO mouse). The reduction of clinically relevant glycosphingolipids lasted 5–6 weeks and then built up again ∼12 weeks after treatment. Consecutive administrations every other week over 3 months yielded a comparable dose-dependent decrease of glycosphingolipids.^66^ Unlike with ERT, no anti-α-Gal A antibody activity was detected following several doses of GLA mRNA, suggesting superior immunological tolerance. Similar promising results were observed in four wild-type non-human primates. After repeated intravenous infusions of human α-Gal A LNP-mRNA, α-Gal A enzyme activity was significantly increased in target tissues, without anti-α-Gal A antibodies or liver function abnormalities.^66^ Overall, these data suggest wide α-Gal A distribution following mRNA administration, providing adequate protein levels in the target organs of Fabry disease (heart and kidneys).

In 2019, DeRosa *et al*.^67^ developed LNPs that accumulate in the liver, enabling the synthesis of supraphysiological α-Gal A enzyme levels, and for the liver to act as a depot for protein translation, endogenous post-translational modification and protein trafficking towards target tissues. High serum protein levels were detected up to 48 h after α-Gal A mRNA LNP injection in mice and marmosets. Compared to ERT (eight weekly doses), the mRNA approach (two monthly doses) yielded comparable decreases in Gb3 and lyso-Gb3 levels, implying that mRNA-based therapy may offer long-term treatment advantages for Fabry disease patients.

Overall, these studies showed that mRNA therapy may enable efficient and sustained synthesis of α-Gal A protein for Fabry disease treatment. The approach appears to be safe, efficient and capable of generating functional protein in multiple species. These findings encourage further investigation and development of mRNA-based treatments for Fabry disease and other lysosomal storage diseases.

### Friedreich’s ataxia

Friedreich’s ataxia (FRDA) is a rare, debilitating, life-shortening, degenerative neurological disorder of autosomal recessive origin, caused by an intronic trinucleotide (GAA) expansion in intron 1 of the frataxin (*FXN*) gene.^68^ Reduced FXN results in progressive pathology in certain cell types, including specific neurons (e.g. sensory neurons in dorsal root ganglia), cardiomyocytes and pancreatic islets. No disease-modifying therapy is available for FRDA, but increased protein levels represent a promising therapeutic avenue.

Nabhan and colleagues^69^ first transfected codon-optimized human FXN mRNA *in vitro* (in 293T cells), leading to recombinant FXN synthesis and mitochondrial processing into mature functional protein (mFXN). Next, they intravenously administered LNP-encapsulated FXN mRNA in adult wild-type mice, demonstrating efficient uptake in hepatocytes and proper maturation of all FXN protein in ∼24 h. Remarkably, the *in vivo* half-life of the recombinant protein in the liver was over 1 week. Finally, they intrathecally administered (via lumbar puncture) the LNP-encapsulated FXN mRNA in adult wild-type mice, yielding recombinant FXN synthesis in the dorsal root ganglia (∼3-fold higher than endogenous control mouse mFXN), which are the primary site of neuropathology in FRDA.^69^

This study is promising for treatment of FRDA and for other neurological disorders with primary neuronal involvement, not secondary to systemic accumulation of toxic metabolites.

## Challenges in treating neurological disorders

Strong rationale and accumulating preclinical evidence highlight mRNA therapy as a treatment for genetic neurological disorders. However, challenges must be addressed. Below, we discuss the greatest barriers to the advancement of mRNA therapeutics in this field: delivery across the blood–brain barrier; repeated dosing; immunogenicity and toxicity; and costs and regulatory issues.

### Delivery across the blood–brain barrier

Therapeutic RNAs are systemically administered using nanosized drug formulations (e.g. LNPs) that enter organs through fenestrated or non-fenestrated capillaries. However, their distribution to specific organs can be hindered by intercellular connections; dense extracellular fibril networks; and non-fenestrated, impermeable vascular endothelial cells.^70^ LNPs cannot cross the blood–brain barrier formed by endothelial cells, tight junctions and adherent processes, complicating the development of mRNA therapies for neurological disorders affecting the CNS and potentially necessitating intrathecal delivery via lumbar puncture or intracerebroventricular administration.^71,72^ Injection into CSF through lumbar puncture has been used to deliver antisense oligonucleotides (ASOs), another type of RNA treatment recently approved for spinal muscular atrophy and amyotrophic lateral sclerosis.^73^ This strategy yields a high therapeutic mRNA concentration in target tissue with minimal circulation outside the CNS. However, it may be challenging to achieve uniform brain targeting with this approach because deep brain regions differ in distance from the CSF. Local delivery to brain structures can be accomplished through direct intraparenchymal administration, with distribution confined to areas near the injection site.^24^ While intracerebroventricular administration can deliver mRNA to neuronal cells and astrocytes of specific brain regions in mouse models, its translation to widespread clinical use is hampered by the complexity and inherent risks associated with neurosurgical procedures.^74,75^

One innovative strategy to cross the blood–brain barrier involves LNPs coated with receptor-specific monoclonal antibodies (mAbs) that target specific epitopes on CNS endothelial receptors, facilitating their transport through receptor-mediated transcytosis following intravenous injection. This strategy, termed the ‘Trojan horse’ approach,^72^ allows LNPs to reach the extracellular space of the CNS. Transferrin receptors are abundantly expressed on the blood–brain barrier, making them suitable ligands for molecule delivery. mAbs can be conjugated to LNPs through a thioether bond between the thiolated mAb and a maleimide moiety of the PEG strands.^76^ ‘Trojan horse’ LNPs have expressed plasmid DNA *in vivo* in the CNS of mice, rats and monkeys.^72^

Natural delivery systems—including extracellular vesicles (EVs), membrane vesicles and exosomes (small EVs)—have been explored as mRNA delivery methods.^77^ Exosomes can be loaded with mRNA through cell transduction before EV isolation or by mixing or electroporation to incorporate target RNA after isolation. EVs are biocompatible and hypo-immunogenic,^77^ and preclinical studies show that they are well-tolerated. Moreover, cell type-specific EVs enable targeted delivery to specific tissues, and EVs from blood cells can cross the blood–brain barrier.

Another proposed strategy for CNS delivery of therapeutic agents is through nasal mucosa absorption, which appears favourable for low-molecular-weight (<1 kDa) and highly lipophilic proteins. Disadvantages include limited achievable concentrations in different CNS regions and poor uptake for high-molecular-weight molecules.^78^

Once LNPs cross the blood–brain barrier, they must be uptaken by neurons and/or glia to exert therapeutic action. Depending on the disease, they may have to target specific CNS cell populations, possibly through cell-targeted LNP-mediated mRNA delivery technologies.^79^ These innovative approaches leverage interactions between tailored LNPs and cellular ligands to target certain CNS populations. Rungta *et al*.^80^ demonstrated that neuronal LNP uptake depended on ApoE, as shown by selective LNP absorption by rat primary neurons in ApoE-supplemented culture media. Future studies focused on refining LNP designs and cell targeting mechanisms could advance therapeutic strategies for neurological disorders.

### Repeated dosing

The need for recurrent dosing hinders the clinical translation of mRNA therapeutics for chronic neurological disorders. Chronic dosage may activate innate immunity, decreasing target protein production, especially in patients who originally completely lacked the protein.^26,81^ Moreover, short intervals between administrations presents practical concerns for many neurological patients, who often have motor and cognitive disabilities. The demand for elevated and durable protein expression has prompted development of various strategies to optimize mRNA content, by minimizing innate immune responses, improving mRNA stability and maximizing translation efficiency (Fig. 1).

Intracellular stability and translational efficacy can be improved by optimizing mRNA structural components—including the 5′ cap, 5′- and 3′-UTRs, coding region and poly(A) tail.^82^ In eukaryotes, the 5′ ends of mRNAs undergo crucial modification, termed capping, wherein a 7-methylguanosine (m7G) cap is added to the first transcribed nucleotide through a 5′-5′ triphosphate bridge (m7GpppN structure).^83^ This cap is pivotal in mRNA function, protection from degradation, favouring splicing processes, improving mRNA transportation and increasing translation efficiency by linking eIF4E (cap-binding protein) and the ribosomal small subunit. A synthetic cap analogue is added during *in vitro* transcription reactions. However, some mRNA remains uncapped due to competition between the cap analogue and GTP nucleotide.^84^ Moreover, conventional cap analogues undergo reverse incorporation of the 5′ cap in 30% of cases.^85^ This led to development of anti-reverse cap analogues (ARCAs) with the hydroxyl group substituted with a methoxy group at the ribose C2 or C3 position. This forces RNA polymerase to initiate transcription using the remaining hydroxyl group, thereby necessitating ARCA incorporation in the forward orientation. All transcripts synthesised with ARCA at the 5′ end are fully translatable, yielding robust stimulatory impact upon translation. Finally, a phosphorothioate-containing ARCA cap analogue was created to provide resistance to mRNA decapping enzyme 2 (DCP2), increasing the mRNA half-life.^86^ New methods have been developed to further improve the capping rate, such as co-transcriptional capping reactions (e.g. CleanCap) and post-transcriptional enzymatic capping through vaccinia virus-capping enzyme (i.e. VCE capping).^87-89^

Another method to enhance mRNA translation and stability is UTR optimization, i.e. inclusion of 5′- and 3′-UTRs containing regulatory sequence elements.^90^ The 5ʹ and 3ʹ UTR sequences modulate the interactions of mRNA with RNA binding proteins, ribosomes and microRNA.^32^ While 5ʹ UTRs should not include start and stop codons, 3ʹ UTRs should contain secondary structure-forming sequences that benefit from the inclusion of different copies of 3ʹ UTRs or ‘fusion UTRs’.^91^

The mRNA should also contain the ORF, encoding the protein’s final amino acid sequence, as an intron-depleted sequence. The preferential and non-random use of certain synonymous codons (human codon usage bias) can be harnessed to optimize the mRNA codon sequence. Strategically modifying the sequence to contain a higher GC:AU ratio yields several advantages, including translation efficiency, enhanced mRNA stability and modulation of protein folding and function.^92^

The discovery that chemically modified nucleosides in uridine moieties can significantly boost protein expression is among the most important developments in mRNA therapies.^19^ It is possible to reduce intracellular TLR recognition and stop mRNA breakdown by replacing uridine with translationally tolerable alternatives (e.g. pseudouridine and *N*^1^-methylpseudourine, m1Ψ). Similarly, converting cytidine to 5-methyl cytidine (m5C) reduces immunogenicity and improves stability and translational efficiency.^93^

The polyadenine tail, poly(A), also controls mRNA stability and translational efficiency by inhibiting premature degradation.^90,94^ In eukaryotes, the poly(A) polymerase independently adds a poly(A) tail, which is then covered by cytosolic poly(A)-binding (PABPC) proteins.^95^ The poly(A) tail influences translation engagement by PABPC-dependent or -independent mechanisms.^96^ In synthetic mRNA, either the poly(A) stretch is encoded in the template DNA, or the mRNA is enzymatically extended by a recombinant poly(A)polymerase in a two-step procedure. The optimal poly(A) tail length is between 120–150 nucleotides, and the 3′ end should not be covered by additional bases.^97^ Heteronucleotidic tails containing both adenine and cytidine have successfully enhanced the protein production rate and stability of synthetic mRNAs for *in vitro* and *in vivo* applications.^98,99^

Self-amplifying mRNA (saRNA) and circular RNA (circRNA) are emerging as promising alternatives to traditional linear mRNA molecules, yielding more protein per coding molecule.^100^ SaRNA is a new technology inspired by positive-sense single-stranded RNA viruses (alphaviruses), substituting the sequence encoding the structural proteins with the gene of interest. Alphaviruses can self-amplify due to several nonstructural viral proteins,^101^ which serve as replicases and duplicate the RNA encoding the structural viral proteins, through RNA-dependent RNA synthesis. Using the intrinsic characteristics of alphaviruses, saRNA can efficiently create greater amounts of a desired protein (20-fold increase compared to conventional approaches), for longer periods of time (20–26 days versus a few days with conventional linear mRNA).^100,101^ However, the longer sequence of saRNA is unfavourable for molecular cloning and protein expression. Additionally, it requires unmodified uridine and presents a double-stranded state in cells, making it more immunogenic than conventional mRNA. Alternatively, nucleoside-modified RNA and an amplifying sequence can be co-transfected as two different molecules, such that the regulatory proteins amplify RNA that can be modified with pseudouridine.^102,103^

Circular RNA creates a closed loop structure shielded from exonuclease activity and from TLR recognition,^104,105^ and functions in nature as a transcriptional regulator, microRNA sponge, or protein template. Compared to linear mRNA, synthetic circRNAs have a longer half-life and are more stable (the absence of free ends lets them dodge recognition by exonucleases). To promote translation on ribosomes, which favour linear mRNA molecules, circular RNAs include internal ribosomal entry site (IRES) sequences (to guide cell type-specific expression) and can be translated without expensive 5′ capping and cumbersome 3′ poly(A) tail modifications.^106,107^

### Immunogenicity and toxicity issues

Although mRNA medicines carry no known substantial hazards and generally display good safety profiles, there is no significant clinical experience with mRNA-based protein replacement therapies.^108^

From the safety perspective, it is crucial to consider mRNA’s immune-activating ability, particularly when systemically injected. The formulation of the mRNA and LNPs determines immunological activation and cytokine release, indicating a need for cautious dose-escalation techniques with modest starting dosages and regular patient monitoring. Additionally, mRNA molecules must be free from contaminants (e.g. dsRNA, truncated species, or DNA-RNA hybrids from DNA digestion), which may activate intracellular detection mechanisms or cellular apoptosis, or increase the cytokine storm risk. Repeated administration regimens carry a risk of developing antibodies against mRNA-encoded proteins, reducing effectiveness. Immune response against the encoded protein may occur if the recipient lacks prior exposure to it, which is also a risk with other gene therapies. Unlike recombinant protein medications, *in vivo*-produced proteins derived from human cells are considered autologous and benefit from proper post-translational modifications, reducing the likelihood of immunological activation. Theoretically, anti-RNA antibodies may develop and start immunological diseases. It may be warranted to perform laboratory tests for antinuclear antibodies and clinical monitoring for autoimmune disorders.

The genotoxic risk seems low compared to conventional DNA-based gene therapies. Direct integration of RNA into double-stranded DNA appears unfeasible. There is no clear evidence of insertional risk of DNA sequences retrotranscribed from exogenous mRNA molecules, except a single observation restricted to tumour cells.^109^ Nevertheless, comprehensive analysis of the genotoxic risk of mRNA therapies is lacking, and studies should address this and additional points (e.g. the impact of exogenous RNA on cells’ epigenetic signatures).^109-111^

Nucleic acid-based medications that directly target or exert indirect immunological effects on the liver can potentially lead to hepatic toxicity. Therefore, it is essential to exercise caution and closely monitor liver enzymes.^112^

Finally, administration of mRNA therapies along with other medications that influence RNA metabolism and translation requires careful consideration. For example, some antibiotics (e.g. aminoglycosides and macrolides) and anticancer drugs (e.g. platinum-based drugs and anthracyclines) may affect RNA metabolism and translation processes in the cells.^39^ While direct interactions between mRNA therapies and these medications are not well-documented, healthcare providers should exercise caution and closely monitor patients when administering these therapies concurrently, especially regarding a possible reduced effect of mRNA-based treatments.

### Costs and regulatory issues

Manufacture of mRNA is fairly straightforward and cost-effective, partly due to the simplified manufacturing processes. Moreover, the same production protocol can be used by changing only the mRNA target sequence.^26^ Conversely, the high cost of DNA-based therapies (approximately $850 000–$3 500 000 per treatment) significantly hinders accessibility and broad application. These costs primarily stem from the manufacturing of AAV for delivery.^113,114^ While the literature includes no direct cost comparisons between mRNA therapies and traditional gene therapies, the lower manufacturing complexities of mRNA suggest potential cost advantages. Kis *et al*.^115^ highlighted the cost-effectiveness and efficiency of non-virally delivered RNA-based vaccines (i.e. mRNA and saRNA), compared to adenovirus-vectored mRNA (AV) platforms. The former are less capital-intensive and more efficient in production than AV. Specifically, saRNA achieves the highest volumetric productivity: 731 million doses per litre per year, compared to mRNA’s 37 million doses and AV’s 154 000 doses. Notably, the quantity of AAV-based therapeutic products required for clinical trials could overcome the current production capacity of industrial facilities, an issue bound to worsen with the growing number of patients to be treated.^33,116^ In a recent analysis, the consulting firm McKinsey underscored the hurdles to scaling up viral vector production, impacting both cost and the speed at which emerging therapies can reach the market.^117^ This lack of scalability raises serious concerns about meeting the demand for large quantities of viral vectors.^113^ An emblematic example of the limitations of DNA-based approaches is alipogene tiparvovec (brand name Glybera®), which was approved to treat lipoprotein lipase deficiency (LPLD), a rare metabolic disorder. Glybera proved commercially unsuccessful due to its high price, high production and maintenance costs, and the rarity of the disorder, and was withdrawn after only 2 years on the market.^118^

Non-viral mRNA delivery may represent a cost-efficient alternative. Synthesis of mRNA is relatively simple and highly reproducible, in contrast to the intricate design and production demands of plasmid DNA required for viral vectors.^119^ Additionally, protein replacement via an mRNA precursor may not be considered a genetic modification from a regulatory perspective, potentially simplifying legal issues surrounding the development of such medicinal techniques.^32^ However, debate over these regulatory issues is ongoing.^120^ Moreover, the costs of licensing patented chemical modifications of mRNA structure and delivery systems must be considered, as they could impact affordability and widespread accessibility to innovative mRNA gene therapies.^113^

## Conclusions and future perspectives

The evidence summarized here supports that mRNA replacement therapy is a promising strategy for treating loss-of-function monogenic neurological disorders. RNA-based medicines avoid several drawbacks of traditional gene therapy methods. Moreover, synthetic mRNA can be produced quickly and affordably, and its production and purification procedures are reliable and conducive to high-throughput methods for drug identification and optimization.

Despite these intrinsic advantages and recent remarkable progress in this field, the establishment of mRNA as a universal therapeutic modality for neurological disorders requires overcoming several issues—including delivery across the blood–brain barrier, cell-specific targeting, repeated dosing, toxicity and unsolved regulatory issues. To surmount these challenges, a multitude of cutting-edge technologies are under development, including various strategies to optimize mRNA structure, create lipid carriers with tissue-targeting abilities and devise efficient *in vivo* delivery systems. By integrating these advancements, we can unlock the full capabilities of biologically targeted mRNA therapeutics, extending beyond their current applications in vaccines and immunostimulatory agents. This holds promise for addressing a wide array of clinical indications and extending the scope of mRNA therapeutics in the field of neurology.

Overall, the potential of RNA-based therapeutics to transform therapeutic procedures in neurological disorders cannot be over-emphasized. The widespread and cost-effective development of RNA-based therapeutics could increase access to affordable treatments for people with loss-of-function neurological disorders that are currently incurable.

## Supplementary Material

awae135_Supplementary_Data
