# Supplementary material for: Unleashing the potential of mRNA therapeutics for inherited neurological diseases
Source: Brain. 2024 Apr 25;147(9):2934–45. doi: 10.1093/brain/awae135 (PMC11969220; doi:10.1093/brain/awae135)
Supplement: awae135_Supplementary_Data [file awae135_supplementary_data.pdf]

Supplementary Table 1. **mRNA therapeutics for monogenic neurological disorders**

Disease (gene)	Therapeutic strategy	Dosing	Preclinical model	Preclinical results	Clinical trials	References
Methylmalonic acidaemia (*MUT*)	IV administration of LNP-encapsulated hMUT mRNA	Every week	Mouse	Hepatic production of functional MUT, reduced plasma methylmalonic acid, improved survival, and increased weight gain	Yes (ongoing phase 1, ID: NCT04899310)	[50,51]
Propionic acidaemia (*PCCA*/*PCCB*)	IV administration of LNP-encapsulated hPCCA and hPCCB (dual mRNA)	Every 3 or 4 weeks	Mouse	Restoration of liver PCC and sustained reduction of primary disease-associated toxins	Yes (ongoing phase 1, ID: NCT04159103)	[53]

Phenylketonuria (*PAH*)	IV administration of LNP-encapsulated human PAH (hPAH) mRNA (LUNAR technology)	Every 3 days	Mouse	Expression of hPAH protein in hepatocytes and reduced plasma phenylalanine	No	59
	IV administration of LNP-encapsulated mouse Pah (MmPah) mRNA	Every 5 days	Mouse	Reduction of phenylalanine in the plasma, liver, and brain	No	60
	IV administration of LNP-encapsulated bacterial PAL mRNA (avPAL) (LUNAR technology)	Single dose	Mouse	Sustained reduction in plasma levels of phenylalanine	No	58

Arginase deficiency (*ARG1*)	IV administration of LNP-encapsulated hARG1 mRNA	Single dose	Mouse	hARG1 protein expression in liver	No	65
	Oral administration of LNP-encapsulated hARG1 mRNA	Every 3 days	Mouse	Hepatic hARG1 expression and activity; reduced levels of plasma ammonia, arginine, glutamine, and guanidinoacetic acid; improved survival	No	66
	Intraperitoneal administration of LNP-encapsulated hARG1 mRNA	Every day	Mouse	Recovery of myelin density and prevention of leukodystrophy	No	68

Fabry disease (*GLA*)	IV administration of LNP-encapsulated hGLA mRNA	Every 2 weeks	Mouse, non-human primates	Enhanced hepatic α-Gal A activity and increased plasma α-Gal A protein level, with substrate reduction	No	71
	IV administration of LNP-encapsulated hGLA mRNA	Every 2 weeks	Mouse, non-human primates	Supraphysiological levels of α-Gal A in liver	No	72
Friedreich's ataxia (*FXN*)	IV or intrathecal administration of LNP-encapsulated hFXN	Single dose	Mouse	Production of mFXN; recombinant human FXN protein in dorsal root ganglia	No	74

IV, Intravenous; PAH, phenylalanine hydroxylase; MUT, methylmalonyl-CoA mutase; PA, propionic acidaemia; PCC, propionyl-CoA carboxylase; PCCA, six alpha subunits; PCCB, six beta subunits; ARG1, Arginase 1; α-Gal A, galactosidase alpha; mFXN, mature functional form of FXN

References

1. Faravelli I, Nizzardo M, Comi GP, Corti S. Spinal muscular atrophy—recent therapeutic advances for an old challenge. Nature Reviews Neurology. 2015;11(6):351-359.
2. Grabowski GA, Zimran A, Ida H. Gaucher disease types 1 and 3: Phenotypic characterization of large populations from the ICGG Gaucher Registry. American journal of hematology. 2015;90:S12-S18.
3. Picache JA, Zheng W, Chen CZ. Therapeutic strategies for Tay-Sachs disease. Frontiers in Pharmacology. 2022;13:906647.
4. Blau N, Van Spronsen FJ, Levy HL. Phenylketonuria. The Lancet. 2010;376(9750):1417-1427.
5. Van Deutekom JC, Van Ommen G-JB. Advances in Duchenne muscular dystrophy gene therapy. Nature Reviews Genetics. 2003;4(10):774-783.
6. McConkie-Rosell A, Finucane B, Cronister A, Abrams L, Bennett RL, Pettersen BJ. Genetic counseling for fragile x syndrome: updated recommendations of the national society of genetic counselors. Journal of genetic counseling. 2005;14(4):249-270.
7. Bertholet AM, Millet AM, Guillermin O, et al. OPA1 loss of function affects in vitro neuronal maturation. Brain. 2013;136(5):1518-1533.
8. de Majo M, Koontz M, Marsan E, et al. Granulin loss of function in human mature brain organoids implicates astrocytes in TDP-43 pathology. Stem Cell Reports. 2023;18(3):706-719.
9. Lopez AD, Murray CC. The global burden of disease, 1990–2020. Nature medicine. 1998;4(11):1241-1243.
10. Chiu ATG, Li J, Chang RSK, et al. Prevalence and healthcare utilization of rare neurological diseases in Hong Kong: 2014–2018. European Journal of Neurology. 2021;28(7):2305-2312.
11. Reinhard C, Bachoud-Lévi A-C, Bäumer T, et al. The European reference network for rare neurological diseases. Frontiers in neurology. 2021;11:616569.
12. Ginn SL, Amaya AK, Alexander IE, Edelstein M, Abedi MR. Gene therapy clinical trials worldwide to 2017: An update. The journal of gene medicine. 2018;20(5):e3015.
13. Kim DH, Rossi JJ. Strategies for silencing human disease using RNA interference. Nature Reviews Genetics. 2007;8(3):173-184.
14. Avci-Adali M, Behring A, Steinle H, et al. In vitro synthesis of modified mRNA for induction of protein expression in human cells. JoVE (Journal of Visualized Experiments). 2014;(93):e51943.
15. Kwon H, Kim M, Seo Y, et al. Emergence of synthetic mRNA: In vitro synthesis of mRNA and its applications in regenerative medicine. Biomaterials. 2018;156:172-193.
16. Wolff JA, Malone RW, Williams P, et al. Direct gene transfer into mouse muscle in vivo. Science. 1990;247(4949):1465-1468.
17. Kariko K, Weissman D. Naturally occurring nucleoside modifications suppress the immunostimulatory activity of RNA: implication for therapeutic RNA development. Current Opinion in Drug Discovery and Development. 2007;10(5):523.
18. Karikó K, Muramatsu H, Ludwig J, Weissman D. Generating the optimal mRNA for therapy: HPLC purification eliminates immune activation and improves translation of nucleoside-modified, protein-encoding mRNA. Nucleic acids research. 2011;39(21):e142-e142.
19. Karikó K, Buckstein M, Ni H, Weissman D. Suppression of RNA recognition by Toll-like receptors: the impact of nucleoside modification and the evolutionary origin of RNA. Immunity. 2005;23(2):165-175.

20. Teufel R, Carralot J-P, Scheel B, et al. Human peripheral blood monuclear cells transfected with messenger RNA stimulate antigen-specific cytotoxic T-lymphocytes in vitro. Cellular and Molecular Life Sciences CMLS. 2005;62:1755-1762.
21. Whittaker JW. Cell-free protein synthesis: the state of the art. Biotechnology letters. 2013;35:143-152.
22. Pardi N, Hogan M, Porter F, Weissman D. mRNAvaccines—a new era invaccinology. Nat RevDrugDiscov 17, 261–279. 2017.
23. Magadum A, Kaur K, Zangi L. mRNA-based protein replacement therapy for the heart. Molecular Therapy. 2019;27(4):785-793.
24. Rohner E, Yang R, Foo KS, Goedel A, Chien KR. Unlocking the promise of mRNA therapeutics. Nature biotechnology. 2022;40(11):1586-1600.
25. Qureischi M, Mohr J, Arellano-Viera E, Knudsen SE, Vohidov F, Garitano-Trojaola A. mRNA-based therapies: Preclinical and clinical applications. International Review of Cell and Molecular Biology. 2022;372:1-54.
26. Qin S, Tang X, Chen Y, et al. mRNA-based therapeutics: powerful and versatile tools to combat diseases. Signal transduction and targeted therapy. 2022;7(1):166.
27. Wang D, Tai PW, Gao G. Adeno-associated virus vector as a platform for gene therapy delivery. Nature reviews Drug discovery. 2019;18(5):358-378.
28. Van Alstyne M, Tattoli I, Delestree N, et al. Gain of toxic function by long-term AAV9-mediated SMN overexpression in the sensorimotor circuit. Nature neuroscience. 2021;24(7):930-940.
29. Huichalaf C, Perfitt TL, Kuperman A, et al. In vivo overexpression of frataxin causes toxicity mediated by iron-sulfur cluster deficiency. Molecular Therapy-Methods & Clinical Development. 2022;24:367-378.
30. Yue Y, Wasala NB, Bostick B, Duan D. 100-fold but not 50-fold dystrophin overexpression aggravates electrocardiographic defects in the mdx model of Duchenne muscular dystrophy. Molecular Therapy-Methods & Clinical Development. 2016;3:16045. doi:10.1038/mtm.2016.45
31. Khorkova O, Stahl J, Joji A, Volmar C-H, Wahlestedt C. Amplifying gene expression with RNA-targeted therapeutics. Nature Reviews Drug Discovery. 2023:1-23.
32. Tavernier G, Andries O, Demeester J, Sanders NN, De Smedt SC, Rejman J. mRNA as gene therapeutic: how to control protein expression. Journal of controlled release. 2011;150(3):238-247.
33. Kowalski PS, Rudra A, Miao L, Anderson DG. Delivering the messenger: advances in technologies for therapeutic mRNA delivery. Molecular Therapy. 2019;27(4):710-728.
34. Gao M, Zhang Q, Feng X-H, Liu J. Synthetic modified messenger RNA for therapeutic applications. Acta Biomaterialia. 2021;131:1-15.
35. Weissman D, Karikó K. mRNA: fulfilling the promise of gene therapy. Molecular Therapy. 2015;23(9):1416-1417.
36. Van Der Werf S, Bradley J, Wimmer E, Studier FW, Dunn JJ. Synthesis of infectious poliovirus RNA by purified T7 RNA polymerase. Proceedings of the National Academy of Sciences. 1986;83(8):2330-2334.
37. Tannous BA, Laios E, Christopoulos TK. T7 RNA polymerase as a self-replicating label for antigen quantification. Nucleic Acids Research. 2002;30(24):e140-e140.
38. Kim Y-K. RNA therapy: rich history, various applications and unlimited future prospects. Experimental & Molecular Medicine. 2022;54(4):455-465.
39. Sahin U, Karikó K, Türeci Ö. mRNA-based therapeutics—developing a new class of drugs. Nature reviews Drug discovery. 2014;13(10):759-780.

40. Schmidt-Wolf GD, Schmidt-Wolf IG. Non-viral and hybrid vectors in human gene therapy: an update. Trends in molecular medicine. 2003;9(2):67-72.
41. Kauffman KJ, Dorkin JR, Yang JH, et al. Optimization of lipid nanoparticle formulations for mRNA delivery in vivo with fractional factorial and definitive screening designs. Nano letters. 2015;15(11):7300-7306.
42. Jung HN, Lee S-Y, Lee S, Youn H, Im H-J. Lipid nanoparticles for delivery of RNA therapeutics: Current status and the role of in vivo imaging. Theranostics. 2022;12(17):7509.
43. Wadhwa A, Aljabbari A, Lokras A, Foged C, Thakur A. Opportunities and challenges in the delivery of mRNA-based vaccines. Pharmaceutics. 2020;12(2):102.
44. Martini PG, Guey LT. A new era for rare genetic diseases: messenger RNA therapy. Human Gene Therapy. 2019;30(10):1180-1189.
45. Zhou X, Cui Y, Han J. Methylmalonic acidemia: Current status and research priorities. Intractable & rare diseases research. 2018;7(2):73-78.
46. An D, Schneller JL, Frassetto A, et al. Systemic messenger RNA therapy as a treatment for methylmalonic acidemia. Cell reports. 2017;21(12):3548-3558.
47. An D, Frassetto A, Jacquinet E, et al. Long-term efficacy and safety of mRNA therapy in two murine models of methylmalonic acidemia. EBioMedicine. 2019;45:519-528.
48. Shchelochkov OA, Carrillo N, Venditti C. Propionic acidemia. 2016;
49. Jiang L, Park J-S, Yin L, et al. Dual mRNA therapy restores metabolic function in long-term studies in mice with propionic acidemia. Nature Communications. 2020;11(1):5339.
50. Van Spronsen FJ, van Wegberg AM, Ahring K, et al. Key European guidelines for the diagnosis and management of patients with phenylketonuria. The lancet Diabetes & endocrinology. 2017;5(9):743-756.
51. Pietz J. Neurological aspects of adult phenylketonuria. Current opinion in neurology. 1998;11(6):679-688.
52. Sabbagh Y, Tenenhouse H, Econs M, Auricchio A. The online metabolic and molecular bases of inherited disease. Mendelian hypophosphatemias New York: McGraw-Hill Companies. 2008;
53. Hennermann JB, Bührer C, Blau N, Vetter B, Mönch E. Long-term treatment with tetrahydrobiopterin increases phenylalanine tolerance in children with severe phenotype of phenylketonuria. Molecular genetics and metabolism. 2005;86:86-90.
54. Diaz-Trelles R, Lee S, Kuakini K, et al. Lipid nanoparticle delivers phenylalanine ammonia lyase mRNA to the liver leading to catabolism and clearance of phenylalanine in a phenylketonuria mouse model. Molecular Genetics and Metabolism Reports. 2022;32:100882.
55. Perez-Garcia CG, Diaz-Trelles R, Vega JB, et al. Development of an mRNA replacement therapy for phenylketonuria. Molecular Therapy-Nucleic Acids. 2022;28:87-98.
56. Cacicedo ML, Weinl-Tenbruck C, Frank D, et al. Phenylalanine hydroxylase mRNA rescues the phenylketonuria phenotype in mice. Frontiers in Bioengineering and Biotechnology. 2022;10:993298.
57. Diez-Fernandez C, Rüfenacht V, Gemperle C, Fingerhut R, Häberle J. Mutations and common variants in the human arginase 1 (ARG1) gene: Impact on patients, diagnostics, and protein structure considerations. Human mutation. 2018;39(8):1029-1050.
58. Müürsepp I, Aibast H, Gapeyeva H, Pääsuke M. Motor skills, haptic perception and social abilities in children with mild speech disorders. Brain and development. 2012;34(2):128-132.
59. Morales A, Sticco KL. Arginase Deficiency. 2018;

60. Asrani KH, Cheng L, Cheng CJ, Subramanian RR. Arginase I mRNA therapy–a novel approach to rescue arginase 1 enzyme deficiency. RNA biology. 2018;15(7):914-922.
61. Truong B, Allegri G, Liu X-B, et al. Lipid nanoparticle-targeted mRNA therapy as a treatment for the inherited metabolic liver disorder arginase deficiency. Proceedings of the National Academy of Sciences. 2019;116(42):21150-21159.
62. Truong B. Human Induced Pluripotent Stem Cell-and mRNA-based Gene Therapy Strategies for Treatment of Arginase Deficiency. University of California, Los Angeles; 2019.
63. Khoja S, Liu X-B, Truong B, et al. Intermittent lipid nanoparticle mRNA administration prevents cortical dysmyelination associated with arginase deficiency. Molecular Therapy-Nucleic Acids. 2022;28:859-874.
64. El-Sayed AS, Shindia AA, Diab AA, Rady AM. Purification and immobilization of L-arginase from thermotolerant Penicillium chrysogenum KJ185377. 1; with unique kinetic properties as thermostable anticancer enzyme. Archives of Pharmacal Research. 2014:1-10.
65. Cortés-Saladelafont E, Fernández-Martín J, Ortolano S. Fabry Disease and Central Nervous System Involvement: From Big to Small, from Brain to Synapse. International Journal of Molecular Sciences. 2023;24(6):5246.
66. Zhu X, Yin L, Theisen M, et al. Systemic mRNA therapy for the treatment of Fabry disease: preclinical studies in wild-type mice, Fabry mouse model, and wild-type non-human primates. The American Journal of Human Genetics. 2019;104(4):625-637.
67. DeRosa F, Smith L, Shen Y, et al. Improved efficacy in a Fabry disease model using a systemic mRNA liver depot system as compared to enzyme replacement therapy. Molecular Therapy. 2019;27(4):878-889.
68. Campuzano V, Montermini L, Molto MD, et al. Friedreich's ataxia: autosomal recessive disease caused by an intronic GAA triplet repeat expansion. Science. 1996;271(5254):1423-1427.
69. Nabhan JF, Wood KM, Rao VP, et al. Intrathecal delivery of frataxin mRNA encapsulated in lipid nanoparticles to dorsal root ganglia as a potential therapeutic for Friedreich’s ataxia. Scientific reports. 2016;6(1):20019.
70. Liu P, Chen G, Zhang J. A review of liposomes as a drug delivery system: current status of approved products, regulatory environments, and future perspectives. Molecules. 2022;27(4):1372.
71. Tanaka H, Nakatani T, Furihata T, et al. In vivo introduction of mRNA encapsulated in lipid nanoparticles to brain neuronal cells and astrocytes via intracerebroventricular administration. Molecular pharmaceutics. 2018;15(5):2060-2067.
72. Pardridge WM. Brain gene therapy with Trojan horse lipid nanoparticles. Trends in Molecular Medicine. 2023;29(5):343-353.
73. McCauley ME, Bennett CF. Antisense drugs for rare and ultra-rare genetic neurological diseases. Neuron. 2023;
74. Piguet F, de Saint Denis T, Audouard E, et al. The challenge of gene therapy for neurological diseases: strategies and tools to achieve efficient delivery to the central nervous system. Human Gene Therapy. 2021;32(7-8):349-374.
75. Atkinson Jr AJ. Intracerebroventricular drug administration. Translational and Clinical Pharmacology. 2017;25(3):117-124.
76. Huwyler J, Wu D, Pardridge WM. Brain drug delivery of small molecules using immunoliposomes. Proceedings of the National Academy of Sciences. 1996;93(24):14164-14169.
77. Li Z, Liu Z, Wu J, Li B. Cell-Derived Vesicles for mRNA Delivery. Pharmaceutics. 2022;14(12):2699.

78. Bahadur S, Pardhi DM, Rautio J, Rosenholm JM, Pathak K. Intranasal nanoemulsions for direct nose-to-brain delivery of actives for CNS disorders. Pharmaceutics. 2020;12(12):1230.
79. Kularatne RN, Crist RM, Stern ST. The future of tissue-targeted lipid nanoparticle-mediated nucleic acid delivery. Pharmaceuticals. 2022;15(7):897.
80. Rungta RL, Choi HB, Lin PJ, et al. Lipid nanoparticle delivery of siRNA to silence neuronal gene expression in the brain. Molecular therapy Nucleic acids. 2013;2(12):e136.
81. Kiaie SH, Majidi Zolbanin N, Ahmadi A, et al. Recent advances in mRNA-LNP therapeutics: immunological and pharmacological aspects. Journal of nanobiotechnology. 2022;20(1):276.
82. Ouranidis A, Vavilis T, Mandala E, et al. mRNA therapeutic modalities design, formulation and manufacturing under pharma 4.0 principles. Biomedicines. 2021;10(1):50.
83. Shatkin A. Capping of eucaryotic mRNAs. Cell. 1976;9(4):645-653.
84. Furuichi Y. Discovery of m7G-cap in eukaryotic mRNAs. Proceedings of the Japan Academy, Series B. 2015;91(8):394-409.
85. Grzela R, Piecyk K, Stankiewicz-Drogon A, et al. N2 modified dinucleotide cap analogs as a potent tool for mRNA engineering. RNA. 2023;29(2):200-216.
86. Jemielity J, Fowler T, Zuberek J, et al. Novel “anti-reverse” cap analogs with superior translational properties. Rna. 2003;9(9):1108-1122.
87. R Kore A, Charles I, Shanmugasundaram M. Organic synthesis and improved biological properties of modified mRNA cap analogs. Current Organic Chemistry. 2010;14(11):1083-1098.
88. Daniel S, Kis Z, Kontoravdi C, Shah N. Quality by Design for enabling RNA platform production processes. Trends in Biotechnology. 2022;
89. Ohno H, Akamine S, Mochizuki M, et al. Versatile strategy using vaccinia virus-capping enzyme to synthesize functional 5′ cap-modified mRNAs. Nucleic Acids Research. 2023;51(6):e34-e34.
90. Jalkanen AL, Coleman SJ, Wilusz J. Determinants and implications of mRNA poly (A) tail size–does this protein make my tail look big? Elsevier; 2014:24-32.
91. Vavilis T, Stamoula E, Ainatzoglou A, et al. mRNA in the Context of Protein Replacement Therapy. Pharmaceutics. 2023;15(1):166.
92. Komar AA. The Yin and Yang of codon usage. Human molecular genetics. 2016;25(R2):R77-R85.
93. Bornewasser L, Domnick C, Kath-Schorr S. Stronger together for in-cell translation: natural and unnatural base modified mRNA. Chemical Science. 2022;13(17):4753-4761.
94. Mauro VP, Chappell SA. A critical analysis of codon optimization in human therapeutics. Trends in molecular medicine. 2014;20(11):604-613.
95. Kühn U, Wahle E. Structure and function of poly (A) binding proteins. Biochimica Et Biophysica Acta (BBA)-Gene Structure and Expression. 2004;1678(2-3):67-84.
96. Thoma C, Bergamini G, Galy B, Hundsdoerfer P, Hentze MW. Enhancement of IRES-mediated translation of the c-myc and BiP mRNAs by the poly (A) tail is independent of intact eIF4G and PABP. Molecular cell. 2004;15(6):925-935.
97. Mockey M, Gonçalves C, Dupuy FP, Lemoine FM, Pichon C, Midoux P. mRNA transfection of dendritic cells: synergistic effect of ARCA mRNA capping with Poly (A) chains in cis and in trans for a high protein expression level. Biochemical and biophysical research communications. 2006;340(4):1062-1068.
98. Balmayor ER. Synthetic mRNA–emerging new class of drug for tissue regeneration. Current opinion in biotechnology. 2022;74:8-14.

99. Li CY, Liang Z, Hu Y, et al. Cytidine-containing tails robustly enhance and prolong protein production of synthetic mRNA in cell and in vivo. Molecular Therapy-Nucleic Acids. 2022;30:300-310.
100. Comes JD, Pijlman GP, Hick TA. Rise of the RNA machines–self-amplification in mRNA vaccine design. Trends in Biotechnology. 2023;
101. Lou G, Anderluzzi G, Schmidt ST, et al. Delivery of self-amplifying mRNA vaccines by cationic lipid nanoparticles: The impact of cationic lipid selection. Journal of Controlled Release. 2020;325:370-379.
102. Beissert T, Perkovic M, Vogel A, et al. A trans-amplifying RNA vaccine strategy for induction of potent protective immunity. Molecular Therapy. 2020;28(1):119-128.
103. Charette M, Gray MW. Pseudouridine in RNA: what, where, how, and why. IUBMB life. 2000;49(5):341-352.
104. Zhang H-d, Jiang L-h, Sun D-w, Hou J-c, Ji Z-l. CircRNA: a novel type of biomarker for cancer. Breast cancer. 2018;25:1-7.
105. Yang Q, Li F, He AT, Yang BB. Circular RNAs: expression, localization, and therapeutic potentials. Molecular Therapy. 2021;29(5):1683-1702.
106. Huang X, Kong N, Zhang X, Cao Y, Langer R, Tao W. The landscape of mRNA nanomedicine. Nature Medicine. 2022;28(11):2273-2287.
107. Sun M, Yang Y. Biological functions and applications of circRNA-next generation of RNA-based therapy. Journal of Molecular Cell Biology. 2023:mjad031.
108. Duan Q, Hu T, Zhu Q, Jin X, Chi F, Chen X. How far are the new wave of mRNA drugs from us? mRNA product current perspective and future development. Frontiers in Immunology. 2022;13:974433.
109. Aldén M, Olofsson Falla F, Yang D, et al. Intracellular reverse transcription of Pfizer BioNTech COVID-19 mRNA vaccine BNT162b2 in vitro in human liver cell line. Current issues in molecular biology. 2022;44(3):1115-1126.
110. Banoun H. mRNA: Vaccine or Gene Therapy? The Safety Regulatory Issues. International Journal of Molecular Sciences. 2023;24(13):10514.
111. Merchant HA. Comment on Aldén et al. Intracellular Reverse Transcription of Pfizer BioNTech COVID-19 mRNA Vaccine BNT162b2 In Vitro in Human Liver Cell Line. Curr. Issues Mol. Biol. 2022, 44, 1115–1126. Current Issues in Molecular Biology. 2022;44(4):1661-1663.
112. Delire B, De Martin E, Meunier L, Larrey D, Horsmans Y. Immunotherapy and gene therapy: New challenges in the diagnosis and management of drug-induced liver injury. Frontiers in pharmacology. 2022;12:786174.
113. Antas P, Carvalho C, Cabral-Teixeira J, de Lemos L, Seabra MC. Toward low-cost gene therapy: mRNA-based therapeutics for treatment of inherited retinal diseases. Trends in Molecular Medicine. 2023;
114. Collins LT, Ponnazhagan S, Curiel DT. Synthetic Biology Design as a Paradigm Shift toward Manufacturing Affordable Adeno-Associated Virus Gene Therapies. ACS synthetic biology. 2023;12(1):17-26.
115. Kis Z, Tak K, Ibrahim D, et al. Pandemic-response adenoviral vector and RNA vaccine manufacturing. npj Vaccines. 2022;7(1):29.
116. De Luca M, Cossu G. Cost and availability of novel cell and gene therapies: Can we avoid a catastrophic second valley of death? EMBO reports. 2023;24(2):e56661.
117. Gaudet D, Stroes ES, Methot J, et al. Long-term retrospective analysis of gene therapy with alipogene tiparvovec and its effect on lipoprotein lipase deficiency-induced pancreatitis. Human gene therapy. 2016;27(11):916-925.

118. Ohlson J. Plasmid manufacture is the bottleneck of the genetic medicine revolution. Drug discovery today. 2020;25(11):1891.
119. Knezevic I, Liu MA, Peden K, Zhou T, Kang H-N. Development of mRNA vaccines: scientific and regulatory issues. Vaccines. 2021;9(2):81.
